# Supplementary material for: HIVprotI: an integrated web based platform for prediction and design of HIV proteins inhibitors
Source: J Cheminform. 2018 Mar 9;10:12. doi: 10.1186/s13321-018-0266-y (PMC5845081; doi:10.1186/s13321-018-0266-y)
Supplement: Supplementary file 2 — Additional file 2. Source code of HIVProtI web server. [file 13321_2018_266_MOESM2_ESM.zip › HIVprotI_Source-code/hivprotI/weka-3-6-6/doc/weka/experiment/SplitEvaluator.html]

SplitEvaluator


---


|  |  |  |
| --- | --- | --- |
| **Overview** \| **Package** \| **Class** \| **Tree** \| **Deprecated** \| **Index** \| **Help** \| **Weka's home** | |  |
| **PREV CLASS**   **NEXT CLASS** | **FRAMES**    **NO FRAMES**     **All Classes** |  |
| SUMMARY: NESTED \| FIELD \| CONSTR \| METHOD | DETAIL: FIELD \| CONSTR \| METHOD |  |


---


## weka.experiment Interface SplitEvaluator

**All Superinterfaces:**: java.io.Serializable

**All Known Implementing Classes:**: ClassifierSplitEvaluator, CostSensitiveClassifierSplitEvaluator, DensityBasedClustererSplitEvaluator, RegressionSplitEvaluator

---

``` public interface SplitEvaluator extends java.io.Serializable ```

Interface to objects able to generate a fixed set of results for
a particular split of a dataset. The set of results should contain
fields related to any settings of the SplitEvaluator (not including
the dataset name. For example, one field for the classifier used to
get the results, another for the classifier options, etc).

Possible implementations of SplitEvaluator:   

- StdClassification results- StdRegression results

**Version:**
:   $Revision: 1.7 $

**Author:**
:   Len Trigg (trigg@cs.waikato.ac.nz)

---

| **Method Summary** | |
| --- | --- |
| `java.lang.Object[]` | `getKey()`             Gets the key describing the current SplitEvaluator. |
| `java.lang.String[]` | `getKeyNames()`             Gets the names of each of the key columns produced for a single run. |
| `java.lang.Object[]` | `getKeyTypes()`             Gets the data types of each of the key columns produced for a single run. |
| `java.lang.String` | `getRawResultOutput()`             Returns the raw output for the most recent call to getResult. |
| `java.lang.Object[]` | `getResult(Instances train, Instances test)`             Gets the results for the supplied train and test datasets. |
| `java.lang.String[]` | `getResultNames()`             Gets the names of each of the result columns produced for a single run. |
| `java.lang.Object[]` | `getResultTypes()`             Gets the data types of each of the result columns produced for a single run. |
| `void` | `setAdditionalMeasures(java.lang.String[] additionalMeasures)`             Sets a list of method names for additional measures to look for in SplitEvaluators. |

**Method Detail**

### setAdditionalMeasures

```
void setAdditionalMeasures(java.lang.String[] additionalMeasures)
```

:   Sets a list of method names for additional measures to look for
    in SplitEvaluators.

    :   **Parameters:**: `additionalMeasures` - a list of method names

---


### getKeyNames

```
java.lang.String[] getKeyNames()
```

:   Gets the names of each of the key columns produced for a single run.
    The names should not contain spaces (use '\_' instead for easy
    translation.) The number of key fields must be constant for a given
    SplitEvaluator.

    :   **Returns:**: an array containing the name of each key column

---


### getKeyTypes

```
java.lang.Object[] getKeyTypes()
```

:   Gets the data types of each of the key columns produced for a single run.
    The number of key fields must be constant
    for a given SplitEvaluator.

    :   **Returns:**: an array containing objects of the type of each key column. The objects should be Strings, or Doubles.

---


### getResultNames

```
java.lang.String[] getResultNames()
```

:   Gets the names of each of the result columns produced for a single run.
    The names should not contain spaces (use '\_' instead for easy
    translation.) The number of result fields must be constant
    for a given SplitEvaluator.

    :   **Returns:**: an array containing the name of each result column

---


### getResultTypes

```
java.lang.Object[] getResultTypes()
```

:   Gets the data types of each of the result columns produced for a
    single run. The number of result fields must be constant
    for a given SplitEvaluator.

    :   **Returns:**: an array containing objects of the type of each result column. The objects should be Strings, or Doubles.

---


### getKey

```
java.lang.Object[] getKey()
```

:   Gets the key describing the current SplitEvaluator. For example
    This may contain the name of the classifier used for classifier
    predictive evaluation. The number of key fields must be constant
    for a given SplitEvaluator.

    :   **Returns:**: a value of type 'Object'

---


### getResult

```
java.lang.Object[] getResult(Instances train,
                             Instances test)
                             throws java.lang.Exception
```

:   Gets the results for the supplied train and test datasets.

    :   **Parameters:**: `train` - the training Instances.: `test` - the testing Instances. **Returns:**: the results stored in an array. The objects stored in the array may be Strings, Doubles, or null (for the missing value). **Throws:**: `java.lang.Exception` - if a problem occurs while getting the results

---


### getRawResultOutput

```
java.lang.String getRawResultOutput()
```

:   Returns the raw output for the most recent call to getResult. Useful
    for debugging splitEvaluators.

    :   **Returns:**: the raw output corresponding to the most recent call to getResut


---


|  |  |  |
| --- | --- | --- |
| **Overview** \| **Package** \| **Class** \| **Tree** \| **Deprecated** \| **Index** \| **Help** \| **Weka's home** | |  |
| **PREV CLASS**   **NEXT CLASS** | **FRAMES**    **NO FRAMES**     **All Classes** |  |
| SUMMARY: NESTED \| FIELD \| CONSTR \| METHOD | DETAIL: FIELD \| CONSTR \| METHOD |  |


---
